# HIV and the Gut Microbiota, Partners in Crime: Breaking the Vicious Cycle to Unearth New Therapeutic Targets

**DOI:** 10.1155/2015/614127

**Published:** 2015-02-22

**Authors:** Kishanda Vyboh, Mohammad-Ali Jenabian, Vikram Mehraj, Jean-Pierre Routy

**Affiliations:** ^1^Chronic Viral Illness Service, McGill University Health Centre, 3650 Saint Urbain, Montreal, QC, Canada H2X 2P4; ^2^Research Institute, McGill University Health Centre, Montreal, QC, Canada H3H 2R9; ^3^Division of Hematology, McGill University Health Centre, 687 Pine Avenue West, Montreal, QC, Canada H3A 1A1

## Abstract

The gut microbiota plays a key role in health and immune system education and surveillance. The delicate balance between microbial growth and containment is controlled by the immune system. However, this balance is disrupted in cases of chronic viral infections such as HIV. This virus is capable of drastically altering the immune system and gastrointestinal environment leading to significant changes to the gut microbiota and mucosal permeability resulting in microbial translocation from the gut into the peripheral blood. The changes made locally in the gut have far-reaching consequences on the other organs of the body starting in the liver, where microbes and their products are normally filtered out, and extending to the blood and even brain. Microbial translocation and their downstream effects such as increased indolamine 2,3-dioxygenase (IDO) enzyme expression and activity create a self-sustaining feedback loop which enhances HIV disease progression and constitute a vicious cycle of inflammation and immune activation combining viral and bacterial factors. Understanding this self-perpetuating cycle could be a key element in developing new therapies aimed at the gut microbiota and its fallout after infection.

## 1. Introduction

The interplay between gut microbiota and the immune system is a complex balance to maintain health and immunity, notably in chronic inflammatory diseases. Here, we review the changes in gut microbiota during HIV infection and the factors which modulate gut microbiota in relation to inflammation in HIV patients. We also discuss the local and systemic impact of the changes in gut microbiota and microbial translocation from the gut into the periphery in HIV infection. Finally, we discuss the potential immunotherapeutic interventions targeting gut mucosal immunity and microbiota to reduce HIV-induced inflammation.

## 2. Gut Microbiota: A Fragile Long-Term Partnership

As humans, we tend to think of ourselves as independent entities; however we have coevolved with billions of microorganisms that have colonized our mucosal tissues and contribute to our host diversity. The interactions between host and microorganism have recently been identified as a two-way street, where host immune pressure and food intake impact the quality of mucosal-associated flora and in turn certain microbes tailor our local and systemic immune system. The oral-gastrointestinal (GI) tract which contains the largest population of microorganisms constitutes the digestive microbiota, better known as gut microbiota. The healthy gut microbiota is composed of a diverse and highly variable population of microbes that include bacteria, viruses, and over 50 genera of fungi [1, 2]. In physiological conditions, the gut microbiota exerts a predominantly positive effect on our immune defenses such as promoting immune cell maturation [3]. In return for providing a niche rich in nutrients, the microbiota provides for us by means of carbohydrate digestion and fermentation, by vitamin production, and most notably by helping our bodies establish gut-associated lymphoid tissue (GALTs) [4].

One of the more common constituents of the gut microbiota is the multiple strains of Lactobacilli, a lactic acid-producing bacterium which is capable of producing lactacin B, a bacteriocidal compound [5]. Lactobacilli are commonly thought of as highly beneficial, so much so that strains tend to be added to different foods labeled as probiotic in hopes of positively affecting the gut microbiota composition. To look at a few examples,* L. acidophilus* interacts with dendritic cells (DCs) to induce production of interleukin-10 (IL-10), an anti-inflammatory cytokine [6]. In addition,* L. paracasei* works from the other end of the spectrum by means of a protease that it encodes which has the ability to degrade highly inflammatory interferon (IFN) *γ*-induced protein 10 (IP-10, CXCL10) [7]. Together, different strains of Lactobacilli are capable of decreasing inflammation in the GI.

The gut microbiota also has diverse effects on cancer development. One group proposed a “driver-passenger” model for colorectal cancer whereby naturally occurring gut microbiota may act as a “driver” creating DNA damage and driving genome instability leading to creation of tumors. “Passenger” or opportunistic bacteria may then take over leading to a dysbiosis of the gut [8]. Despite potentially triggering colorectal cancer, another group showed that the gut microbiota may also be key in cancer treatment [9]. Cyclophosphamide (CTX), a DNA-alkylating chemotherapy agent, is dependent on a healthy gut microbiota to properly impact the polarization of splenocytes into Th17 cells, which play a key role in maintaining the integrity of mucosal immunity by secretion of IL-17 [10]. Indeed, when CTX is used in germ-free mice, or mice on antibiotic treatments, a reduced elicitation of Th17 cells was found [11].

One of the more established functions of the gut microbiota is prevention of various diseases. By outcompeting pathogenic microorganisms for food and space, the gut microbiota is able to check pathogenic growth and prevent damage to the host [12, 13]. However, some viral infections have been known to use the microbiota to their advantage. Mouse mammary tumor virus (MMTV), a retrovirus, is capable of coating itself in lipopolysaccharides (LPS) derived from the gut microbiota and interacting with pattern recognition receptor toll-like receptor 4 (TLR4) on myeloid cells [14]. The subsequent production of IL-10 contributes to successful MMTV infection via induction of immune tolerance [14]. In addition, poliovirus uses the gut microbiota as well by binding to LPS to promote infection resulting in a more severe clinical course [15]. By using the gut microbiota to their own advantage, viruses such as poliovirus and MMTV are capable of circumventing immune detection and elimination in favour of enhanced replication.

## 3. HIV and the Digestive Tract: A Land of Opportunity

The GALT, in particular CD4+ T cells residing in the GALT, is one of the main sites in HIV infection which constitute a long-term reservoir site even in patients receiving successful antiretroviral therapy (ART) [16]. Whatever the route of infection, mucosal regions house a rich microbiota which alters the infectivity of the target cells. Once infection has occurred, HIV rapidly depletes CD4+ T cells from the GALT as a larger percentage of these cells express elevated level of CCR5, the coreceptor for cellular entry, compared to peripheral blood [17]. Indeed, in an experimental infection of macaques by simian immunodeficiency virus (SIV), a rapid decrease of 90% of CD4+ T cells in the GALT was observed within 2 weeks of infection [18].

The other hallmark of HIV infection is persistent immune activation which makes CD4+ T cells more susceptible to infection, thus creating a vicious cycle by increasing production of IFN-*γ* [19], IL-6 [20], IP-10 [21], and indoleamine2,3-dioxygenase (IDO) [22]. CD4+ T cell destruction associated with immune activation in the gut leads to high levels of CD8+ T cell infiltration and epithelial cellular damage. In addition, HIV-infected cells are known to display an altered expression of microRNAs (miRNAs) in which multiple miRNAs are downregulated [23]. As miRNAs in the GI can also be affected by the microbiota [24, 25], it is entirely likely that HIV creates changes to the GI miRNA profile as well. In the GI tract, Mucosal barrier damages disrupt the integrity of the epithelial tissue and favor microbial translocation into the circulating blood [26]. This “leaking GALT” in addition to HIV has been linked to the development of acquired immunodeficiency syndrome (AIDS) [27]. ART has the ability to partially reconstitute this loss of CD4+ T cells in the gut, but only to roughly 50% when compared to noninfected controls [28]. One of the most significant consequences to the GALT caused by HIV is the drastic decrease of Th17 cells. There is also an increase in immunosuppressive regulatory T cell (Treg) frequency in the GALT which is influenced by the levels of IDO [22]. This shift in the balance of Treg and Th17 cells in favor of Tregs leads to increased mucosal permeability and microbial translocation and therefore further fuels immune activation [29].

## 4. The Importance of the Tryptophan Pathway: A Crossroad between Microbes and Host

IDO is an immunomodulatory enzyme found in dendritic cells (DC) and macrophages which breaks down Tryptophan (Trp) into Kynurenine (Kyn) [30–32]. IDO is known to be induced by IFN-*γ* in response to inflammatory signals [33]. In addition, Tryptophan 2,3-dioxygenase (TDO), a hepatic enzyme, is highly similar to IDO, which also acts on the Kyn pathway [34, 35]. TDO may also be found in the placenta, testis, and brain after stimulation [35–37]. Enhanced immunosuppressive Kyn production by IDO and/or TDO plays a harmful role in cancers and viral infections including HIV infection [22, 29, 38, 39]. Kyn inhibits T cell proliferation [40, 41] while another IDO catabolite, quinolinic acid, is linked to neurodegenerative diseases including AIDS dementia complex [42]. It is known that monocyte derived-DCs specifically expressing IDO promote Treg expansion and that the IDO induction in these DCs can be achieved by the HIV transactivator protein Tat [43, 44]. Furthermore, our team has recently shown that increased IDO enzyme activity and Kyn production are linked to the imbalance of Th17/Treg and microbial translocation in chronic HIV infection [29]. In untreated HIV infection, IDO levels were found to be elevated and were correlated with the high levels of immune activation. After several years of continuous successful ART, these levels decreased, approaching what is seen in healthy subjects [29]. Interestingly, an enrichment of a gut microbiota subset which has the capacity of catabolizing Trp through the IDO pathway was found in HIV-infected subjects [45].

## 5. HIV and Gut Microbiota: Partners in Crime Enhancing Immune Activation in a Stepwise Process

### 5.1. Local Effects

#### 5.1.1. The Gastrointestinal Tract

The alteration of Th17/Treg balance in the GALT induced by HIV leads to microbial translocation of commensal and pathogenic bacterial products into the blood stream resulting in a generalized and persistent immune activation [46]. However, there were also changes to the types and amounts of bacteria that comprise to microbiota. An in-depth analysis of the changes in microbiota of HIV-infected patients was assessed by Vujkovic-Cvijin et al. In their study, the total bacterial load and amount of diversity appeared to be similar across infected and uninfected groups; however HIV viremic patients had microbiota communities distinctly enriched in Proteobacteria, most notably of the family Enterobacteriaceae which includes known pathological microbes such as* Salmonella, Escherichia, *and* Shigella *[45]. In fact, these pathological microbes tend to be the cause of bacteremia in advanced HIV-infected patients [47]. Viremic patients also displayed a decrease in* Bacteroides* and* Alistipes,* which are depleted in inflammatory bowel disease [48]. The particular enrichments and depletions in viremic HIV patients were found to be linked to a decrease in Th17 cells in gut biopsies as well as an increase in immune activation and correlation with IDO activity and IP-10 plasma levels as a trustable marker of HIV disease progression [45]. The link between IDO activity and the microbiota appears to be a self-sustaining feedback loop, which encourages pathological microbe growth. Multiple bacteria enriched in HIV viremic patients in Vujkovic-Cvijin's et al. study have enzymatic homologs of IDO, which are capable of producing Kyn from Trp. The initial assault from HIV to the gut causes inflammation, which may in turn create a microenvironment more suitable to pathologic bacteria. This bacterial community may be capable of outcompeting its beneficial counterpart by way of Kyn production through IDO, and, once established, they are capable of producing Kyn which further fuels their growth.

However, some ART-treated patients exhibited microbial communities highly similar to viremic patients, while others were much more similar to healthy subjects. The diversity may be an indication of clinical outcome or could indicate that the microbiota recovery time is variable. In line with this hypothesis, a recent study by Lozupone et al. looked at bacterial variance during ART [49]. They examined HIV-infected patients who were untreated or had been on ART for varying lengths of time. The study showed that genera of bacteria that are elevated in HIV-infected patients* versus* healthy subjects such as* Peptococcus* decreased over time spent on ART to levels approaching that of healthy subjects [49]. Pérez-Santiago et al. showed related results in a cohort of HIV infected men on successful ART [50]. Indeed, they demonstrated an association between enriched levels of* Lactobacillales* and preserved immune function as indicated by decreased microbial translocation, lower T cell proliferation, and higher percentages of CD4+ T cells in the gut and periphery [50].

Lactobacilli are clearly important for regulating and maintaining physiological gut immunity, a concept which was explored by Zelante et al. in a mouse model [51]. Indeed, Lactobacilli, specifically* L. reuteri*, are capable of catabolizing Trp into indole-3-aldehyde (IAld) when there is an excess of nutritional Trp and IDO activity is low. IAld is then capable of stimulating natural killer (NK) cells via aryl hydrocarbon receptor (AhR) to produce IL-22 which controls the gut microbiota, ensuring a diverse ecosystem [51]. However, in cases where IDO activity is elevated due to the migration of IDO-expressing DCs to gut mucosa, Trp is preferentially broken down into immunosuppressive Kyn. Higher levels of Kyn and the subsequent expansion of Tregs create a tolerogenic environment where normal commensals like* Candida albicans* can become pathogenic creating candidiasis. Interestingly, the same study showed that administration of oral IAld to mice with mucosal candidiasis restored IL-22 production by NK cells and decreased the candidiasis [51]. This distinctive use of Trp by Lactobacilli may in part account for its association with better clinical outcomes in HIV by way of limiting Kyn production and may represent an important strategy for future treatments.

#### 5.1.2. The Liver Firewall

Recently, Balmer et al. helped elucidate the role of the liver in the control of microbial translocation using a mouse model [52]. In their study, livers of healthy mice did not show any signs of containing microbes. However, once the gut epithelial cells were breached, microbes gained access to underlying vasculature, which drains directly into the hepatic portal vein [52]. Mice challenged with* E. coli* alone did not have any detectable bacteria in the liver but after inducing experimental intestinal inflammation,* E. coli* was consistently found in the liver. Once microbial products reach the liver, Kupffer cells, specialized hepatic macrophages, are capable of clearing the bacterial challenge. In the case of liver tissular insults, mice showed a drastic reduction in bacterial clearance [52].

Since the initiation of ART treatment, patients have shown increased survival and that survival has led to a rise in non-AIDS conditions all related to immune activation that affects kidney, cardiovascular organs, and liver [53]. HIV induces hepatic damages via multiple mechanisms. First, the damage can occur directly through infection of Kupffer cells [54]. The liver is further damaged by inflammation, favored by microbial translocation. LPS in the portal vein system is capable of activating Kuppfer cells, leading to a release of inflammatory cytokines and perpetuating the continued inflammation and therefore hepatic damages [53]. Liver damage can further be exacerbated by alcohol abuse, obesity, metabolic syndrome, and ART hepatotoxicity. HIV is also capable of accelerating the development of liver cirrhosis in patients coinfected with HCV [55]. Under viral infection conditions that increase immune system inflammation, the increased microbial translocation is linked to a decrease in the liver's ability to clear bacteria [56]. Epidemiological evidence indicates that a cohort of patients displaying nonalcoholic fatty liver disease or steatohepatitis showed evidence of serum IgG and IgA against intestinal commensal microbes which signifies that compartmentalization of the gastrointestinal microbiota is compromised in liver disease due to the failure of the hepatic vascular firewall [52].

### 5.2. Systemic Effects

#### 5.2.1. Circulating Blood

HIV infection is a major cause of microbial translocation where bacterial products egressing the gut by the portal vein cannot be fully cleared by the Kupffer cells in the liver leading to microbes and their products being present in peripheral blood. Levels of microbial translocation can be measured by sCD14, the soluble form of CD14, released into the circulation by monocytes upon microbial product stimulation [57]. In HIV viremic patients, sCD14 is elevated but, once patients are treated with ART, these levels decrease to a level similar to healthy individuals [58]. IDO enzymatic activity also follows this trend [29]. Another soluble inflammatory marker which is linked to IDO activity is soluble CD40 ligand (sCD40L) as CD40-CD40L signaling is known to be key in IDO induction. sCD40L is mainly produced by activated T cells, platelets, and B cells and its plasma levels are increased in chronic HIV infection [59]. As part of the TNF-receptor superfamily, engagement of CD40 and CD40L, in the presence the HIV envelope protein gp120, can sensitize DCs for apoptosis [60]. Our group has recently reported that sCD40L is able to stimulate Treg expansion and differentiation, and, most notably, production of Kyn through IDO resulting in microbial translocation [61].

IDO can also be used to predict disease outcomes independently of viral load and CD4+ T cell counts. In a Ugandan cohort of HIV-infected patients, higher IDO activity was strongly associated with higher HIV RNA copies and low CD4+ T cell counts in absence of ART. Following ART initiation, IDO levels remained predictive of low CD4+ T cell recovery and increased mortality [62]. Furthermore, the same group identified IDO levels to be associated with neurocognitive disorders [63].

#### 5.2.2. The Brain

IDO produced at local gut mucosal sites and circulated in the peripheral blood affects multiple organs in multiple ways, including the brain. Activated monocytes are capable of trafficking the virus into the central nervous system where the infection is mainly perpetuated by infected macrophages [64]. In fact, it is not only infected cells that can cause complications. Blood-brain barrier endothelial cells can synthesize Kyn after immune activation [65]. In mice, activation of IDO leads to inflammation-associated depression. This induction is mediated in part through the viral protein Tat which synergizes with IFN-*γ* already present due to inflammation [66]. Furthermore, high circulating levels of IDO in HIV patients are associated with depression [67] and are found in HIV-associated dementia [68]. In line with this, it has been shown that sCD40L is also involved in cerebral inflammation and dementia in HIV-infected patients [36, 37, 69, 70].

## 6. Perspective for New Immunotherapeutic Targets

**Figure 1:**
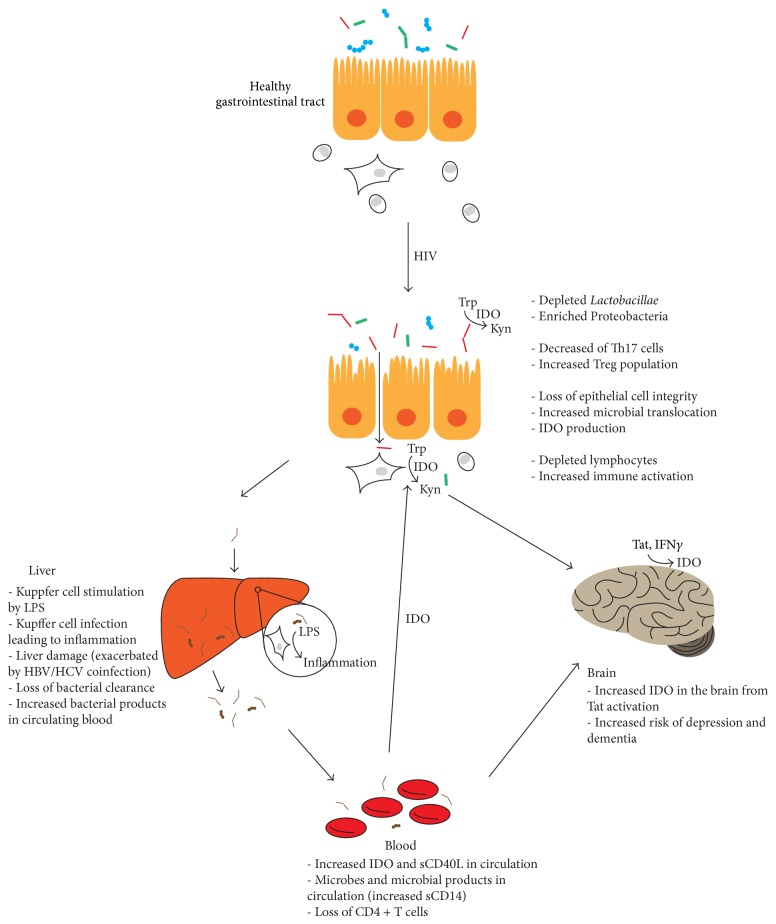
The vicious cycle of HIV infection. HIV infection has immediate effects in the gut where lymphocytes are depleted and damage to the endothelium allows for microbial translocation. Microbiota, when not cleated by the liver, go on to have systemic effects most notably through IDO production which is capable of creating a vicious cycle of inflammation.

The human gut microbiota is complex and deeply intertwined with the immune system, which makes it one of the many factors involved in HIV infection. During HIV infection, the microbiota is affected on a local level in the gastrointestinal tract, which creates changes to our immune system. The alterations to immune system favoring inflammation lead to increased microbial translocation which is normally cleared in the liver, except in cases of liver damage or when this translocation persists for long periods of time. The systemic effects of the microbiota can be explained by the production of IDO, which occurs at the level of the gut and also at multiple sites including the brain. IDO and its immunosuppressive catabolites are further capable of altering the immune system by enhancing Treg populations and downregulating Th17 populations, creating a vicious circle (Figure 1). The topic of intervention in relation to the microbiota is not new and includes targeting the GI biological, immune, and mechanical barrier [64]. However, targeting factors outside of the GI tract may also be beneficial.

All this makes IDO, TDO, CD40L (an upstream inducer of IDO), and the microbiota targets during HIV treatment to improve the immune system as summarized in Table 1. Such attempt used 1-methyl-tryptophan (1-MT) in the brain of CX3CL1−/− mice after challenges of LPS [71]. CX3CL1−/− mice normally display persistent neural inflammation and depressive-like behavior upon LPS challenge but with 1-MT, a competitive inhibitor of IDO, these effects were abrogated only 72 H after challenge [71]. Similar results were seen in another mouse model using 1-MT to promote clearance of HIV-infected macrophages in the brain, an environment simulating HIV encephalitis, where administration of the drug caused infected macrophages to decrease by almost 90% [72]. 1-MT was also used in an SIV model using rhesus macaques on ART. Although Kyn levels remained high, suggesting 1-MT was not fully effective against IDO activity, macaques with unsuccessful ART displayed reduced viral load [73]. However, in a more recent study of 1-MT in rhesus macaques with SIV on ART treatment, there was no effect found on inflammation, viral RNA in blood, or gastrointestinal tissue [74].

IDO may be a potential target not only to treat HIV infection, but also in the prevention of infection. In HIV-exposed seronegative female commercial sex workers, cervical mononuclear cells were shown to have much lower levels of IDO than HIV-infected individuals [75]. One possible avenue in HIV prevention may lie in understanding the “immune quiescent” nature of seronegative sex workers immune system [76].

Another treatment possibility is to act on the microbiota directly. Probiotics and prebiotics help support and grow the microbiota and have been used in different diseases with gastrointestinal inflammation [77]. Probiotics consist of microorganisms, frequently Lactobacilli, that are taken with the aim of positively influencing the host microbiota and therefore health. Prebiotics on the other hand are indigestible food ingredients such as inulin that aim to promote microbiota associated with good health. When used together, one study found that pro- and prebiotics increased CD4+ T cell reconstitution and functionality while a meta-analysis found that probiotics improved infant growth and protected against CD4+ T cell loss [78, 79]. New studies have also begun looking into Sevelamer as a treatment for microbial translocation and subsequent inflammation. Sevelamer is a phosphate-binding drug already shown to decrease blood levels of LPS in cases of chronic kidney disease [80]. Like 1-MT, Sevelamer has conflicting results. Indeed, in an SIV model, Sevelamer decreased microbial translocation while also decreasing inflammation and immune activation [81]. However a study on nontreated HIV-infected patients showed a lack of any significant changes to microbial translocation, inflammation, or immune activation [82].

Although not directly aimed at the microbiota, our group was instrumental in a study using IL-7 as a treatment meant to restore gut immunity and integrity. IL-7 is known to induce gut epithelial cells to produce IL-7, while an absence of gut microbiota is known to decrease IL-7 [83]. After IL-7 administration, Patients showed increased CD4+ and CD8+ T cells, as well as an increase in gut-homing lymphocytes (*α*4*β*7+ T cells). Patients also displayed a decrease in sCD14 indicating an improvement in the gut barrier integrity [84].

## 7. Concluding Remarks

Research has only begun to scratch the surface of how our microbiome fully influences HIV infection. However, it is clear that a complex interplay between gut microbiota and altered immune system mediated by the virus contributes to disease progression and immunodeficiency. Therefore, design and implementation of new and combinatory immunotherapeutic strategies which target both gut microbiota and host immunosuppressive mechanisms could represent novel additions to current ART treatments to reduce generalized immune activation and inflammation as a consequence of HIV/microbiota partnership.

## Figures and Tables

**Table 1:** Selected studies targeting the gut microbiota or subsequent downstream effects.

Therapeutic target	Drug	Study details	Result	Reference
IDO	1-MT	CXCL1−/− mice	Decreased activation in the brain and decreased depressive behaviour	[71]
	1-MT	Mice with injections to the brain of HIV-infected macrophages	Increased CD8+/IFN-*γ*+ T cells in the periphery and an 89% decrease in HIV-infected macrophages in the brain	[72]
	1-MT	SIV in rhesus macaques on ART	No change to T cell counts or activation, viral load, or Trp metabolism	[74]
	1-MT	SIV in rhesus macaques on ART	Only partial effect on IDO activity and significant drop in viral load for macaques with unsuccessful ART	[73]

Pro-/prebiotics	Pro and prebiotics	SIV in pigtail macaques on ART	Enhanced reconstitution and functionality of CD4+ T cells and increased frequency of GI tract APCs	[78]
	*Bifidobacterium lactis *	Meta-analysis of formula supplementation in HIV-infected infants (>6 months)	Improved infant growth and protection against CD4+ T cell loss	[79]

Sevelamer	Sevelamer	Acute SIV infection in pigtail macaques	Drug bound LPS in the gut, drastically decreased inflammation and immune activation, and slightly decreased viral replication	[81]
	Sevelamer	HIV patients not receiving ART	No significant change to microbial translocation, inflammation, or immune activation, but significant decrease in LDL cholesterol	[82]

IL-7	Recombinant human IL-7	HIV patients on successful ART	Increased CD4+ and CD8+ T cells, increased a4b7 T cells, and decreased sCD14	[84]
